# ROS production, intracellular HSP70 levels and their relationship in human neutrophils: effects of age

**DOI:** 10.18632/oncotarget.2856

**Published:** 2014-12-03

**Authors:** Elena I. Kovalenko, Anna A. Boyko, Victor F. Semenkov, Gennady V. Lutsenko, Maria V. Grechikhina, Leonid M. Kanevskiy, Tatyana L. Azhikina, William G. Telford, Alexander M. Sapozhnikov

**Affiliations:** ^1^ Shemyakin and Ovchinnikov Institute of Bioorganic Chemistry RAS, Moscow, Russia; ^2^ Centre of Gerontology of Russian State Medical University, Moscow, Russia; ^3^ National Cancer Institute, National Institute of Health, Bethesda, USA

**Keywords:** Aging, neutrophils, reactive oxygen species, heat shock proteins, HSP70, cell stress

## Abstract

ROS production and intracellular HSP70 levels were measured in human neutrophils for three age groups: young (20-59 years), elders (60-89 years) and nonagenarians (90 years and older). Elders showed higher levels of spontaneous intracellular ROS content compared with young and nonagenarian groups, which had similar intracellular ROS levels. Zymosan-induced (non-spontaneous) extracellular ROS levels were also similar for young and nonagenarians but were lower in elders. However, spontaneous extracellular ROS production increased continuously with age. Correlation analysis revealed positive relationships between HSP70 levels and zymosan-stimulated ROS production in the elder group. This was consistent with a promoting role for HSP70 in ROS-associated neutrophils response to pathogens. No positive correlation between ROS production and intracellular HSP70 levels was found for groups of young people and nonagenarians. In contrast, significant negative correlations of some ROS and HSP70 characteriscics were found for neutrophils from young people and nonagenarians. The observed difference in ROS and HSP70 correlations in elders and nonagenarians might be associated with an increased risk of mortality in older individuals less than 90 years old.

## INTRODUCTION

Aging is usually described as a complex multifactor biological process involving various molecular and functional alterations. Excessive generation of free radicals, especially reactive oxygen species (ROS), together with impaired resistance to cell stress was proposed to play a general role in acceleration of the aging process [1-3]. According to another view, excessive ROS production as well as molecular damage accumulation may accompany aging but does not drive it. Activation of cellular growth and metabolic signaling pathways in mature organisms can lead to aging development and life shortening [4].

Alterations in immune system functioning may also play an important role in human aging. Aged persons are often characterized by a state of chronic inflammation, termed “inflamm-aging” [5]. One feature of this phenomenon is persistent low-grade activation of immune cells, particularly neutrophils that constitute the largest fraction of leukocytes in human organism. Despite a short lifespan these cells play an important role in antibacterial defense. They rapidly migrate to the site of infection, destroy pathogens and initiate inflammatory responses by means of phagocytosis of the pathogens, ROS generation in membrane NADPH oxidase complex, release of cytosolic granules containing proteolytic enzymes, and cytokine/chemokine production. One of the reasons for chronic inflammation may be connected with prolongation of lifespan of activated neutrophils in damaged tissues [6]. It is also known that neutrophil dysfunction is involved in the pathogenesis of many diseases, some of which are associated with age [7, 8].

Numerous findings have shown that neutrophils are affected by aging and may contribute to immunosenescence. Many functions of neutrophils have been shown to be compromised in the elderly. Chemotaxis, phagocytosis, intracellular killing and NET formation all decline with age [9-11]. Spontaneous neutrophil apoptosis is unchanged or slightly increased and cytokine-mediated delay of neutrophil apoptosis is disturbed in aged subjects [12]. Impaired anti-oxidant mechanisms or signal transduction alterations in neutrophils of the elderly people may contribute to the shorter lifespan of these cells [13, 14]. However, the presence of long-lived neutrophils in elderly donors was demonstrated recently [15]. Most findings have demonstrated that ROS production in response to stimulus declines in the elderly [16]. At the same time, spontaneous ROS production of neutrophils may increase with age, participating in chronic inflammatory processes more common in aged persons [17, 18].

One mechanism of cell protection from the adverse consequences of ROS action is provided by highly conserved heat shock proteins (HSPs), ubiquitously expressed intracellular stress proteins [19, 20]. These molecular chaperons are involved in correct protein folding and utilization and prevent protein aggregation, providing cell resistance to stress [21]. The HSP70 family consists of several highly homologous proteins, including constitutively expressed Hsc70 and inducible Hsp70. The highly regulated intracellular localization of HSP70 family members plays an essential role in normal cell functions and in protection of cells from unfavorable conditions [22]. Under certain conditions intracellular HSP70 proteins can be translocated to the plasma membrane or be released from the cells forming extracellular pool of these proteins circulating in the body.

Stress resistance is often associated with extension of lifespan. It has been suggested that the impairment of HSP-dependent mechanisms to maintain protein homeostasis could be a factor contributing to aging [23]. This theory has stimulated a series of studies of age-related changes of HSP70 expression. Most data show that intracellular and serum HSP70 levels were dependent on age. In particular, basal intracellular HSP70 level in blood mononuclear cells was shown to increase in elderly donors [24]. In contrast, the ability of these cells to express HSP70 in response to stress stimulus together with other types of stress responses declined in old age [25]. Concentration of circulating HSP70 in human serum has also been reported to decrease with aging [26, 27]. In mammals and birds higher intracellular levels of heat shock proteins were found in longer-lived organisms [28]. At the same time, low levels of serum Hsp70 were shown to be associated with longevity in humans [29]. Nevertheless, data concerning intracellular content and stress-induced expression of HSP70 in neutrophils are still inconsistent and incomplete. Moreover, there is almost no information about age-associated changes of HSP70 intracellular levels in these cells as well as their capacity to release these proteins. It is noteworthy that spontaneous ROS production in neutrophils from aged people correlated with HSP70 blood plasma levels [17].

Thus, neutrophils being a plentiful source of both intracellular and extracellular ROS may play a significant role in organism aging. It is unclear whether HSP70 is involved in controlling the process of ROS generation in neutrophils. In this work we have analyzed production of ROS and HSP70 content in neutrophils as well as the relationships of these parameters in three age groups of human individuals: 20-59 (young), 60-89 (elders) and 90 and more (nonagenarians) years of age. We demonstrated that both spontaneous and zymosan-induced ROS production changed with age. Some ROS parameters in the group of nonagenarians (90 year and older) were similar to the parameters registered in the group of young volunteers in contrast to the group of elders. Only HSP70 expression associated with heat treatment of neutrophils was found to be age-dependent. Correlation analysis applied to HSP70 and ROS production parameters has revealed multiple relationships between intracellular HSP70 levels and ROS production in neutrophils for different age groups. The positive correlation found only in the group of elders suggests a role for intracellular HPS70 in maintenance of appropriate ROS responses of neutrophils in this group.

## RESULTS

### Spontaneous and induced ROS production in neutrophils from young, old and nonagenarian donors

We initially analyzed age-dependent alterations of ROS generation in human neutrophils using the cells isolated from donors of three age groups: 20-59 (young), 60-89 (elders) and 90 years and older (nonagenarians). Intensive production of ROS is one of the markers of activated neutrophils. It accompanies phagocytosis and secretory degranulation. On the other hand, even freshly isolated neutrophils without any additional stimuli can produce considerable amounts of ROS reflecting their activation history. We analyzed ROS production induced by opsonized zymosan as a characteristic of functional response of neutrophils to a putative pathogen. Before measuring zymosan-induced activation of neutrophils we estimated their spontaneous ROS production. Two methods were applied for spontaneous ROS production assessment: labeling with the cell permeable fluorescent probe DCFH-DA (DCF) for cytometric detection of intracellular ROS, and luminol-amplified chemiluminescence (LAC) as a standard approach to evaluation of extracellular ROS production by phagocytes [30]. LAC was also used to analyze zymosan-induced oxidative burst.

**Figure 1:**
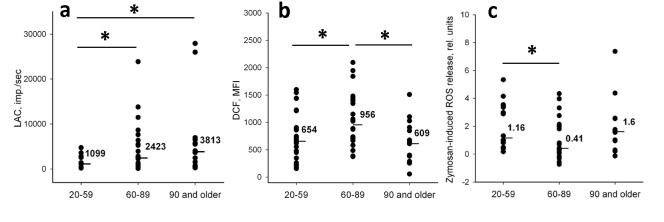
Relative levels of ROS generation in neutrophils isolated from donors of different age (a) Spontaneous ROS production registered by LAC method (ROS_LAC spont_). (b) Spontaneous intracellular ROS generation registered with DCF probe (ROS_DCF spont_); mean fluorescence intensities (MFI) are presented. (c) ROS production stimulated with opsonized zymosan (ROS_LAC ind_). Line bars and numeric values are medians calculated for each group. Significance established at p < 0.05 is shown by asterisk.

Levels of spontaneously produced ROS detected by LAC (ROS_LAC spont_) in neutrophils of elders and nonagenarian donors were significantly higher than the ROS_LAC spont_ levels in neutrophils of young donors (Figure 1a). Spontaneous intracellular ROS generation measured by DCF (ROS_DCF spont_) was also significantly increased in the elder group (Figure 1b). However, ROS_DCF spont_ levels in neutrophils of nonagenarians were similar to the levels in young donors in contradiction with the results obtained for ROS_LAC spont_. Correlation analysis showed no significant relationship between spontaneous ROS production detected in neutrophils by DCF and LAC in all age groups indicating that the methods discriminated ROS of different kinds and/or produced from different sources.

We then estimated age-dependent changes in ROS production induced in neutrophils by zymosan (ROS_LAC ind_). This parameter was calculated for each donor as a ratio of the difference between maximal (ROS_LAC max_) and initial levels of ROS production to the initial level [ROS_LAC ind_ = (ROS_LAC max_ - ROS_LAC spont_)/ROS_LAC spont_]. It should be mentioned that ROS_LAC max_ levels were reached between 10 and 20 min after zymosan addition, depending on the donor. We did not find a significant difference between ROS_LAC ind_ in nonagenarians and young group. At the same time ROS_LAC ind_levels in elders were significantly lower than in the young donors (Figure 1c). So, functional response of neutrophils did not decline in the nonagenarians in contrast to the elders.

### Intracellular HSP70 levels in different age groups and effect of hyperthermia on HSP70 expression

**Figure 2:**
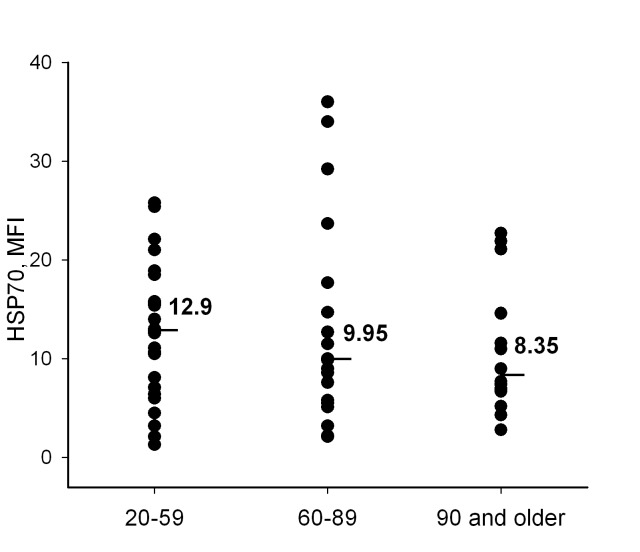
Intracellular content of HSP70 (HSP70) measured in neutrophils by flow cytometry with anti-HSP70 antibody BRM-22 in different age groups Line bars and numeric values present medians calculated for each group.

Cell stress resistance mediated by HSP70 has been proposed as an important anti-aging factor. HSP70 expression in neutrophils in different age donor groups was therefore measured. To estimate intracellular content of the protein we labeled neutrophils with anti-HSP70 antibody (BRM-22, Sigma), recognizing both constitutive (Hsc70) and inducible (Hsp70) forms of HSP70, stained with secondary fluorochrome-conjugated antibody and analyzed them by flow cytometry. We did not reveal any significant age-dependent alterations in HSP70 content (HSP70_basal_) in the cells (Figure 2). Nevertheless, the insignificant decrease in HSP70_basal_ median in neutrophils from the older donor groups might indicate that a more significant difference might be detected in a future study with larger sampling.

**Figure 3:**
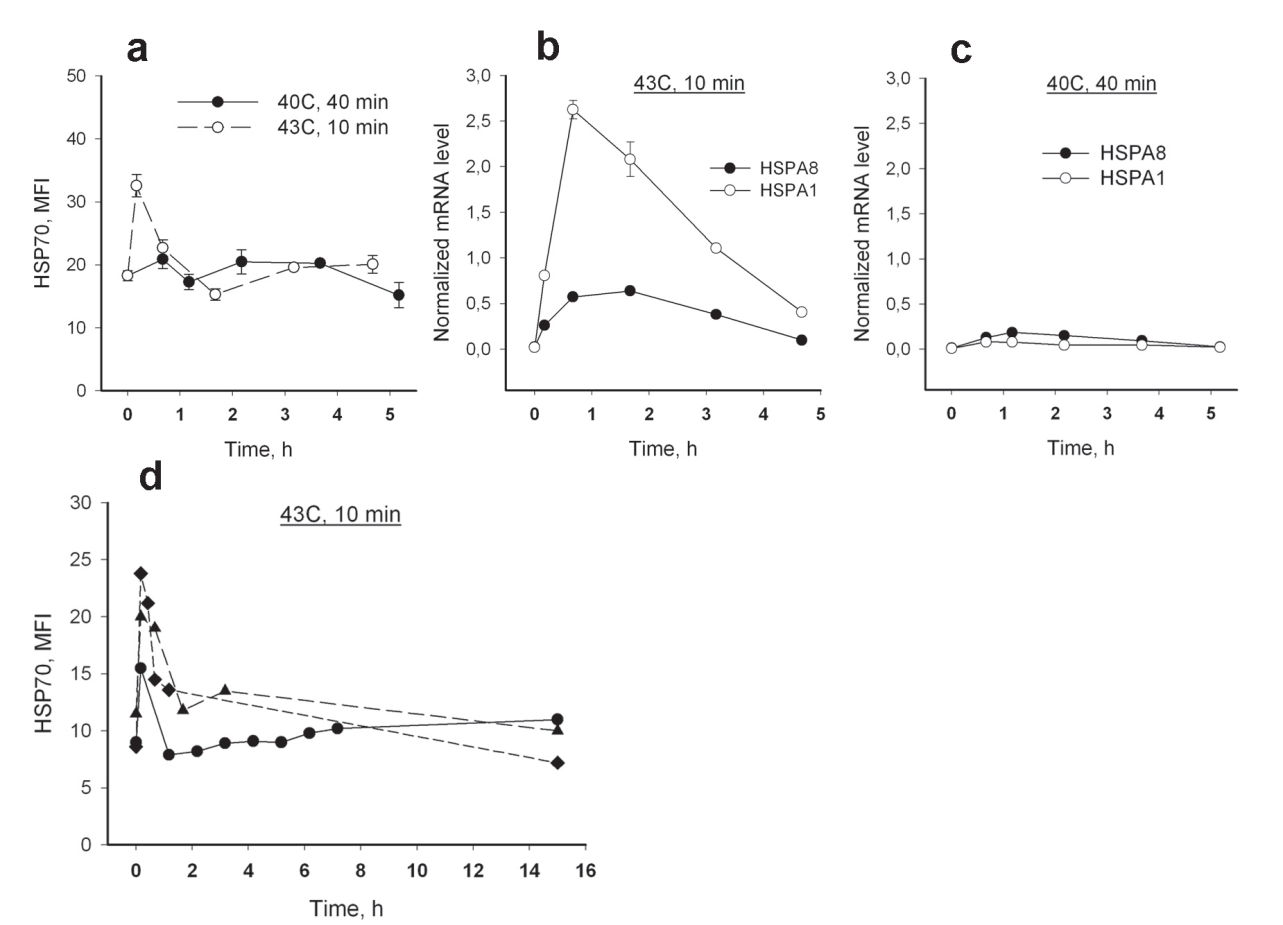
Dynamics of HSP70 response to hyperthermia in human neutrophils (a) Intracellular HSP70 levels following heat treatment; 1 - 40°C, 40 min; 2 - 43°C, 10 min. (b, c) Induction of *HSPA1* and *HSPA8* mRNA synthesis in neutrophils by heat treatment at 40°C, 40 min (b) and 43°C, 10 min (c); mRNA levels were normalized by b-actin. Data presented in A, B and C are the results of the same experiment with neutrophils isolated from a 22 year old donor. (d) Kinetics of intracellular level of HSP70 detected for 15 h in response to heat shock at 43°C, 10 min, obtained in experiments with neutrophils from three donors.

We then analyzed effect of heat shock, the classic stress-inducing microenvironmental condition, on HSP70 expression in neutrophils from the three age groups. Two models of *in vitro* cell hyperthermia, 40 min at 40°C and 10 min at 43°C, were applied to induce a HSP70 stress response in neutrophils. Changes in intracellular HSP70 levels in response to the hyperthermic stress were then measured using the described above immunolabeling protocol. No significant increase in intracellular levels of HSP70 was observed during 4.5 h after heat treatment except a brief increase in HSP70 level immediately after treatment in the cell samples subjected to 43°C heating (Figure 3a). However, mRNA synthesis of both inducible (*HSPA1B*) and constitutive (*HSPA8*) forms of HSP70 did increase in neutrophils between 0.5 h and 3 h after heat treatment for the 43°C regimen. A rise of mRNA synthesis was not seen with the 40°C regimen (Figure 3b, c,). No increase in HSP70 intracellular content was detected at later time points (more than 4.5 h incubation) after neutrophil treatment under either condition (Figure 3d). A considerable decrease in neutrophil numbers in the cell cultures after 15 h incubation connected with neutrophil apoptosis did not allow us to collect the data for appropriate statistics of HSP70 levels at the later time points.

**Figure 4:**
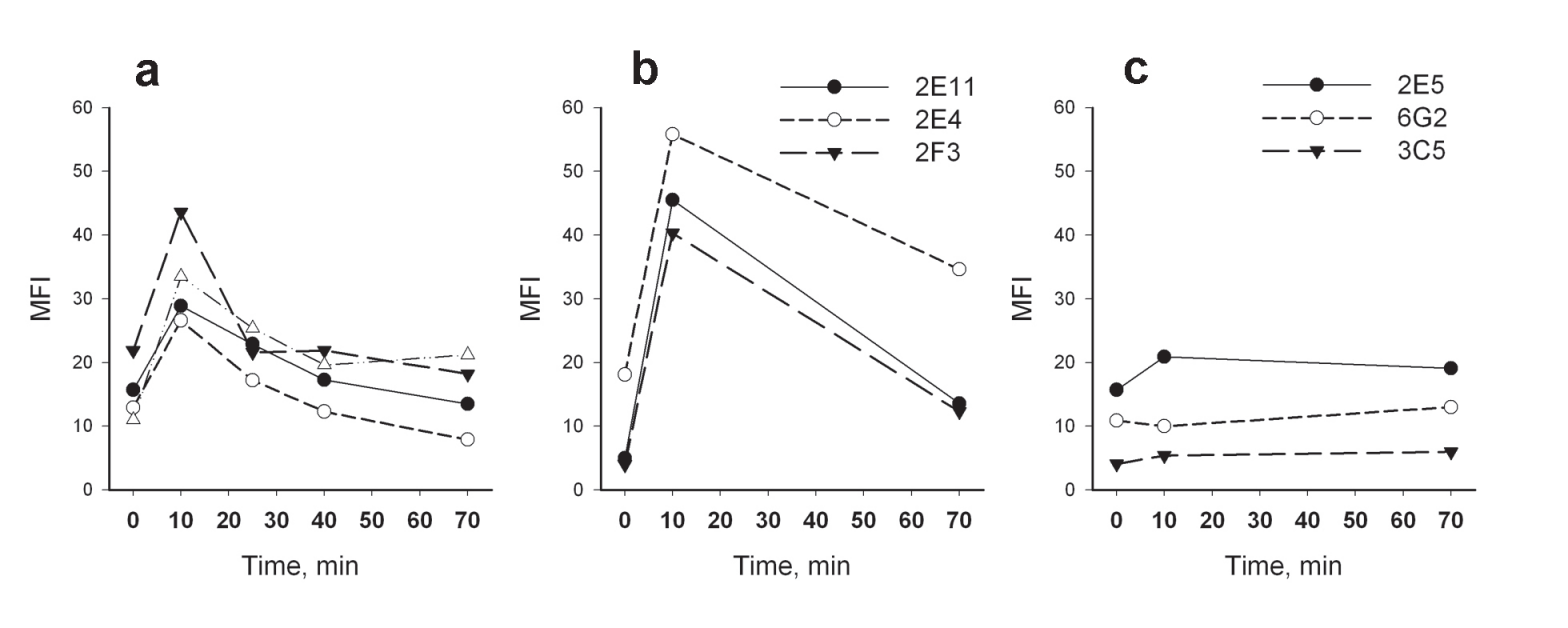
Dynamics of intracellular level of HSP70 after heat shock (43°C, 10 min) analyzed by flow cytometry using different HSP70-specific antibodies (indicated in legend) (a) HSP70 dynamics measured with anti-HSP70 antibody BRM22 (4 donors of ages from 25 to 91 years). (b) HSP70 dynamics recorded with antibodies interacting with *C*-terminal domain of HSP70. (c) HSP70 dynamics recorded with antibodies interacting with *N*-terminal domain of HSP70.

Significant differences in HSP70 intracellular level dynamics were found for two heat treatment regimes used in this study. A biphasic pattern of HSP70 dynamics was observed during first hour after hyperthermia when neutrophils were treated at 43°C for 10 min (hereinafter designated as heat shock); no such pattern of the reaction was observed with the cell treatment at 40°C for 40 min (Figure 3a). An increase of intracellular HSP70 levels was detected immediately after the heat shock termination; then a decrease of the HSP70 levels was observed within next 15-30 min (the typical dynamics curves are presented in Figure 4a).

We next focused on the two-phase pattern of intracellular HSP70 expression connected with immediate effect of heat shock on HSP70 levels as measured by flow cytometry. Two groups of monoclonal antibodies to HSP70 were used (three types of antibodies in each) displaying specificity to *N*- or *C*-domain of HSP70 [31]. Surprisingly we did not detect any increase of HSP70 level after heat shock using antibodies to the *N*-end part of HSP70 responsible for binding with nucleotides. In contrast, antibodies recognizing the *C*-end substrate-binding domain of HSP70 (SBD) showed an increase in HSP70 levels (Figure 4b, c). These results suggested that elevation of HSP70 intracellular level immediately after heat shock was mediated by increased availability of the protein epitopes for interaction with antibodies, rather than an increase of the total protein concentration in the cell. It is important to note that pre-incubation of neutrophils with protein synthesis inhibitor cycloheximide (100 μM, 30 min) did not change the pattern of heat shock dependent intracellular HSP70 dynamics (data not shown) indicating that the observed rapid increase in HSP70 level was not associated with the synthesis of the proteins *de novo*, but may have been due to changes of the molecule conformation. We hypothesize that the conformational changes resulted in the increase of HSP70 level observed immediately after heat shock was connected with the exposure of antibody-binding epitopes of SBD due to dissociation of substrate molecules from HSP70 under the heat shock conditions. From this point of view the observed HSP70 increase after heat shock may indirectly show the amount of HSP70 engaged in functional interactions with client proteins before heat shock.

**Figure 5:**
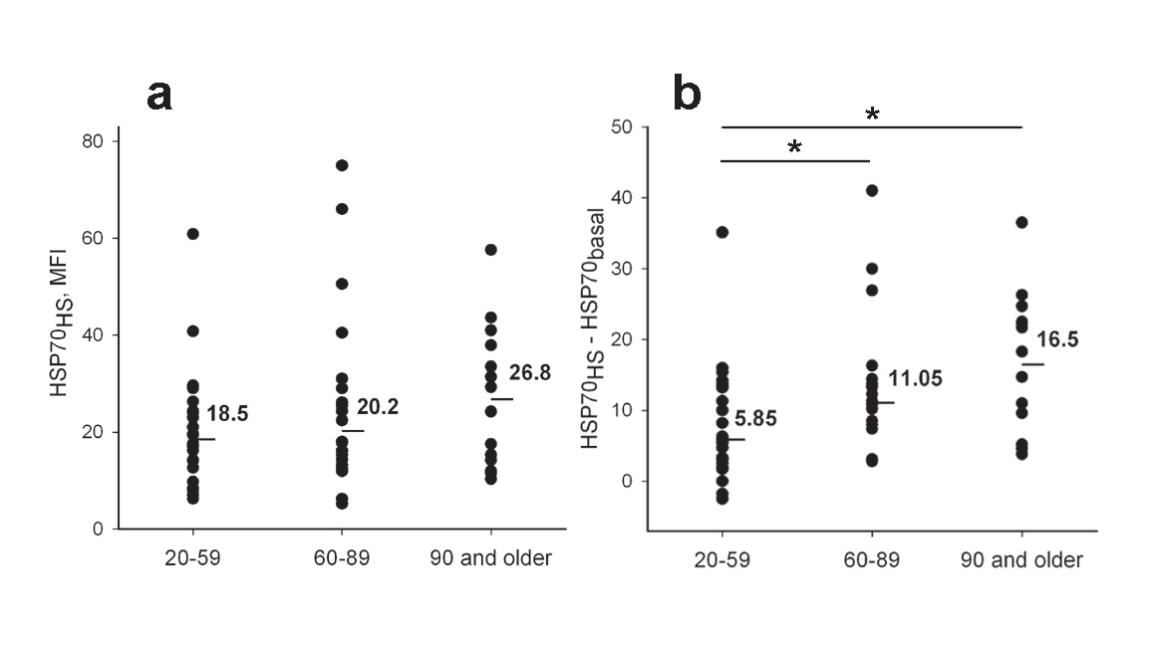
Comparison of heat shock induced elevation of intracellular HSP70 levels in neutrophils from different age groups (a) HSP70 levels recorded immediately after heat shock (HSP70_HS_) (b) HSP70 level increase (ΔHSP70_HS_) calculated by subtraction of HSP70_basal_ from HSP70_HS_. Line bars and numeric values present medians calculated for each group. Asterisks indicate significant difference between the groups (p < 0.05).

Next we examined and compared heat shock-induced elevation of intracellular HSP70 levels in different age groups. Like basal levels (HSP70_basal_), the HSP70 levels measured immediately after heat shock (HSP70_HS_) did not differ significantly between young, elder and nonagenarian donors (Figure 5a). However, the difference between HSP70_HS_ and HSP70_basal_ (ΔHSP70_HS_) for each person were shown to be significantly increased with age (Figure 5b).

### Correlation analysis of HSP70 and ROS parameters in neutrophils of different age groups

Heat shock proteins are engaged in cell protection from damaging ROS action by preventing accumulation of denaturated proteins. The next task of the work was to study relationships between ROS production and intracellular HSP70 levels in neutrophils by correlation analysis. Taking into consideration that most of parameters analyzed in the study were shown to be age-dependent, we compared the relationships in young, elderly and nonagenarian age groups. Several values for both ROS production (ROS_LAC spont_, ROS_DCF spont_, ROS_LAC ind_) and intracellular HSP70 (HSP70_basal_, HSP70_HS_ and ΔHSP70_HS_) were analyzed (Table 1).

**Table1:** Correlation analysis of intracellular HSP70 and ROS production in neutrophils in different age groups (r – correlation coefficient, p – significance value)

Parameter 1	Young donors	Elders	Nonagenarians	Parameter 2
HSP70_basal_	r = –0.086, p = 0.698	r = –0.063, p = 0.834	r = –0.319, p = 0.258	ROS_DCF spont_
	**Negative** **r = –0.634, p = 0.014**	r = 0.082, p = 0.778	r = 0.273, p = 0.377	ROS_LAC spont_
	r = 0.220, p = 0.435	**Positive** **r = 0.615, p = 0.024**	r = –0.329, p = 0.284	ROS_LAC ind_
HSP70_HS_	r = –0.033, p = 0.880	r = –0.203, p = 0.513	**Negative** **r = –0.61, p = 0.020**	ROS_DCF spont_
	**Negative** **r = –0.719, p = 0.003**	r = 0.297, p = 0.313	r = –0.182, p = 0.557	ROS_LAC spont_
	r = –0.073, p = 0.797	**Positive** **r = 0.692, p = 0.008**	r = –0.070, p = 0.817	ROS_LAC ind_
ΔHSP70_HS_	r = 0.029, p = 0.896	r = –0.217, p = 0.484	**Negative** **r = –0.626, p = 0.016**	ROS_DCF spont_
	r = –0.007, p = 0.976	r = 0.396, p = 0.173	**Negative** **r = –0.671, p = 0.015**	ROS_LAC spont_
	r = –0.420, p = 0.129	**Positive** **r = 0.560, p = 0.044**	r = 0.301, p = 0.329	ROS_LAC ind_

Several significant correlations between the ROS and HSP70 parameters were revealed in all age groups suggesting that regulatory relationships might exist between intracellular HSP70 and ROS production in human neutrophils. However it seems unlikely that the relationships are direct, as they do not remain constant with age. Moreover, in different age groups both negative and positive correlations between HSP70 and ROS were found.

In the group of young donors HSP70_basal_ was negatively correlated with ROS_LAC spont_ (Table 1) indicating an association of the lowest spontaneous LAC-detected extracellular ROS production with the highest intracellular HSP70 content in neutrophils. Since there was no correlation between ROS_LAC spont_ and ΔHSP70_HS_ in this group, this negative correlation was not connected with “hidden” HSP70 portion displayed after heat shock. This probably indicated the level of HSP70 engaged in functional interaction with intracellular client proteins. This relationship disappeared with age; we did not found any correlation between HSP70_basal_ and ROS_LAC spont_ in older donor groups.

One of striking characteristics of HSP70–ROS relationships was revealed in the group of elders. Strong positive correlations were found between ROS_LAC ind_ and all parameters of intracellular HSP70 analyzed in this study (Table 1). Thus, in this age group high HSP70 content in neutrophils is associated with high ROS production induced by opsonized zymosan, considered as a protective immune response. So, in the age group between 60 and 89 years, when stimulated ROS production in neutrophils is significantly declined, increased expression of HSP70 can promote adequate ROS response of these cells to a pathogen, probably thanks to chaperone properties of the protein. Importantly, in the group of nonagenarians in which zymosan-induced ROS production was not changed comparing with the young group, no significant correlations between ROS_LAC ind_ and HSP70 parameters were found, similar to the group of young donors.

The relationship in which ΔHSP70_HS_ levels were negatively correlated with parameters of spontaneous ROS generation (registered by both DCF and LAC) was found only in the group of nonagenarians. Presumably, the accumulation of HSP70 interacting with their substrates in neutrophils is functionally associated with decreased spontaneous ROS production in this age group. No significant correlation was revealed for ΔHSP70_HS_ with any ROS production parameters in the young donor group.

Thus, based on the correlation analysis carried out in our study several age-dependent types of relationships between intracellular HSP70 and ROS production in neutrophils can be suggested. However, mechanisms underlying the revealed HSP70–ROS parameter associations need to be further investigated.

## DISCUSSION

Neutrophils use ROS for the destruction of pathogens. Intensive ROS production accompanies many processes in neutrophils including phagocytosis, response to pro-inflammatory stimuli and NET formation [32, 33]. In spite of diverse roles in cell signal cascades and gene expression, ROS also exert deleterious effects in the cells because of the oxidative modification of biological molecules. An intrinsic feature of neutrophils is a multi-level system of protection directed at elimination of ROS excess and reduction of harmful consequences of damaging ROS action. Intracellular HSP70 participates in this system by chaperon activity. Age-associated increase of ROS generation and decline of anti-inflammatory, antioxidant or repairing systems may lead to elevation of amount of misfolded and aggregated proteins and a loss of this protective activity [34]. These phenomena are widely considered as the main causes of aging. From another point of view, over-stimulated signaling pathways related to cell growth and nutrient sensing, particularly mammalian target of rapamycin (mTOR)-associated pathways, and interplay of these pathways with ROS may lead to aging [35].

The present study confirms that ROS generation in neutrophils is dependent on age. Neutrophils in elderly donors had less effective functional ROS response to a stimulus whereas their spontaneous ROS production increased with age (Figure 1). In general, these results concur with published data on age-dependence of ROS production in neutrophils [16, 17]. The delayed kinetics and decreased magnitude of ROS response in neutrophils was described earlier in elders [9, 36]. High levels of spontaneous ROS production detected by LAC in elders and nonagenarians described in this work (Figure 1a) could be interpreted as such delayed response of neutrophils to previous stimuli. Increased spontaneous ROS generation can contribute to the low-grade inflammatory condition, or “inflamm-aging” status, reported for aged volunteers [18]. On the other hand, the high spontaneous ROS levels observed in nonagenarians do not correspond to the suggestion that increased ROS generation is the reason for life shortening.

Interestingly, correlation analysis demonstrated a discrepancy between spontaneous ROS generation levels registered by DCF or LAC (ROS_DCF spont_ and ROS_LAC spont_, respectively). Apparently, the main contribution to the ROS_LAC spont_ is connected with NADPH-oxidase-mediated extracellular ROS production associated with the process of neutrophil activation. The level of intracellular ROS production measured by flow cytometry (ROS_DCF spont_) may be connected with activity of other sources of ROS independent of NADPH-oxidase, in particular with oxidative phosphorylation in mitochondria. Respectively, ROS generation from distinct sources could be regulated differently. The absence of any correlation between ROS_DCF spont_ and ROS_LAC spont_ argues for this interpretation. At the same time, different forms of ROS might be detected by cytometric and chemiluminescence methods. Since fluorescence of DCF-DA is assumed to be proportional to the concentration of hydrogen peroxide in cells [37], various types of ROS including superoxide anion production may be measured non-specifically by luminol-dependent chemiluminescence [38].

We have shown that ROS_DCF spont_ levels in nonagenarians, in contrast to ROS_LAC spont_ levels, were similar to the levels in young group and significanly lower than in elders (Figure 1b). So, age-dependent elevation of extracellular ROS production does not imply a mandatory increase in intracellular ROS in neutrophils. The difference in ROS_DCF spont_ levels between elders and nonagenarians might indicate that “antioxidant shield” is compromised in the elder group but remains undisturbed in nonagenarians where intracellular ROS are scavenged better. Another possibility is that some stress-mediated or activating intracellular events in neutrophils of elders resulted in excessive ROS generation. In its turn, the increase in intracellular ROS levels may lead to activation of ROS-dependent signaling pathways in neutrophils.

Excess of ROS may lead to intracellular molecular impairments in conditions with failed cell protection systems, in particular with deficit of protective heat shock proteins. We have analyzed possible age dependence of intracellular content of HSP70 in neutrophils. Flow cytometric analysis of intracellular HSP70 in intact neutrophils did not reveal any significant difference between the three age groups of donors (Figure 2). For further analysis we used a model with heat treatment of neutrophils *in vitro*. It is known that heat shock evokes two types of effects on the cells. Direct effects involve conformational changes of proteins, changes in interactions between proteins and alterations in activity of proteins as a result of various chemical modifications. Secondary effects become apparent as activation of certain signaling pathways and induction of heat shock protein expression for subsequent protection of cells. However in neutrophils we could not detect a pronounced induction of HSP70 protein accumulation by heating the cells at 40°C or at 43°C, despite an observed increase in gene expression at the second higher temperature (Figure 3). It is interesting that mRNA of both inducible and constitutive forms of HSP70 increased after the treatment. It is possible that the mechanism of protection involving accumulation of HSP70 following heat shock is not easily detectable in such short-lived cell population as neutrophils. Nevertheless, this does not exclude a protective role of HSP70 permanently expressed in neutrophils, particularly taking into account that these cells actively produce ROS and should be well armed against oxidative stress.

Using a series of monoclonal antibodies against HSP70 we next demonstrated the differences in heat shock induced dynamics of intracellular HSP70 levels detected with antibodies recognizing either the N-terminal part or the C-terminal part of HSP70 molecule (Figure 4). A heat shock associated increase in intracellular HSP70 levels measured with antibodies specific to the C-terminal part of HSP70 containing the substrate-binding domain was not connected with HSP70 synthesis *de novo* but was related to conformational changes in the HSP70 molecule. This was due to an increase in availability of the SBD structure for interactions with the antibodies. Detailed information about conformational alterations in HSP70 molecule associated with the change of a client protein was published recently [39, 40]. Presumably, the difference between initial and heat shock induced intracellular HSP70 levels (ΔHSP70_HS_) was related to the numbers of HSP70 molecules involved in active interaction with substrates and released from the molecular complexes upon the cell heating. Importantly, this is the only parameter between HSP70 parameters studied in this work that demonstrated dependence on age (Figure 5). ΔHSP70_HS_was found to increase in both elders and nonagenarians. It is known that aging is associated with increasing concentration of aggregated and mis-folded proteins [41]. All these proteins need HSP70 assistance in cells. Possibly ΔHSP70_HS_ level indirectly determines the amount of the impaired proteins interacting with HSP70 in neutrophils. Cell stress might result in a replacement of the target proteins. From this point of view, the increased ΔHSP70_HS_ in aged individuals argues for effective HSP70-mediated cell protection in old age.

The main focus of this work was to determine if there was any close relationship between ROS generation and intracellular HSP70 expression in neutrophils. To address this question we performed correlation analysis of HSP70 and ROS parameters. A number of the correlations (both positive and negative) have been found (Table) which implies that such relationships might exist. Some other findings support this suggestion. It was shown earlier that HSP70-mediated heat tolerance prevented inhibition of NADPH oxidase and superoxide dismutase activity caused by heat shock in phagocytes [42]. Spontaneous ROS production by neutrophils was demonstrated to be negatively associated with HSP70 in plasma [17]. However, until now there is no clear mechanism proposed for these relationships.

The correlations between intracellular HSP70 and ROS production we observed in neutrophils showed significant age-dependent differences (Table). Importantly, none of the relationships in groups of old donors (elders and nonagenarians) were similar to the correlations found in the group of young people evidenced an essential effect of aging on the relationships analyzed in our work.

In young donors, the low spontaneous extracellular ROS production (ROS_LAC spont_), which can be interpreted as a normal state in resting neutrophils, was associated with high level of intracellular HSP70 (Table). Mechanistically, this can be explained in two ways: 1) intracellular level of HSP70 decreases following an increase of ROS generation; 2) initial low HSP70 level provokes somehow an elevation of ROS_LAC spont_ which we attribute to NADPH-oxidase-mediated ROS production. Although our data favor neither hypothesis, some arguments can be made in support of the first one. In spite of the fact that HSP70 can contribute to late stage anti-oxidant protection, these proteins also might be subjected to the destructive action of ROS. HSP70s have been shown to stabilize the lysosome membrane [43]. ROS-mediated carbonylation of HSP70 with subsequent cleavage by calpain was suggested as a mechanism of lysosomal rupture associated with oxidative stress [44]. Other possibility is that stress-induced release of HSP70 associated with the lysosomal compartment mediates the decrease of intracellular HSP70 [45]. However it is not clear whether these processes occur in neutrophils. It should be noted that in nonagenarians spontaneous ROS production was negatively correlated with ΔHSP70_HS_ levels, itself age-dependent. The biological significance of this association needs further studies.

It is noticeable that elders were the only age group where positive correlations, notably correlations between HSP70 levels (HSP70_basal_, HSP70_HS_ and ΔHSP70_HS_) and zymosan-induced ROS production, were found. Obviously, sufficient amounts of intracellular HSP70 preserved the ROS response of neutrophils to pathogens that was decreased in general within the elders. This suggests an important role for HSP70 in the maintenance of adequate reaction of neutrophils to danger stimuli.

Of particular interest to this study is previous studies demonstrating that characteristics of neutrophils of very old donors (over 80-90 years old) including chemotaxis, phagocytosis, expression of adhesion molecules, intracellular ROS levels, apoptosis rate and antioxidant activities show less difference with young people than with slightly younger old donors in the 60-70-years old range [46-48]. These observations have led to the conclusion that long lived individuals can be considered as a model of successful aging [49]. In this work we found several important differences between elderly volunteers and nonagenarians. Some of characteristics measured in the nonagenarian group were more similar to the young donor group than to the elders. Particularly, levels of zymosan-induced ROS_LAC ind_ produced in neutrophils of elderly people were lower than in the groups of both nonagenarians and young volunteers (Figure 1c). This suggests that neutrophil-associated immune defense may function better in nonagenarians in comparison with the elderly. The highest intracellular spontaneous ROS production in neutrophils was revealed in the group of elders suggesting that these cells are at the risk of oxidative stress. No positive correlation between HPS70 and induced ROS response levels was found in nonagenarians in contrast to elders. Basing on the successful aging model we assume that the noticed set of characteristics specific only for elders do not relate to longevity.

Discrepancies in the correlations found in elders and nonagenarians indicated different mechanisms of HSP70-ROS interplay in these age groups supporting that the relationship was not direct. The dissimilarity of patterns of ROS-HSP70 association and ROS levels in neutrophils for different age groups may be explained basing on hyperfunction theory of aging. In this concept, unintentional continuation of developmental growth of a mature organism is accepted as the main factor causing aging. On the cell level, mechanism of aging is connected with growth signaling, in particular through mTOR-centric pathways, leading to the cell over-stimulation, i.e. hyperfunction [4, 50]. Chronic inflammation mediated largely by activated neutrophils may be considered as a typical example of hyperfunction on the organismal level [5]. Neutrophils actively use mTOR pathway for regulation of inflammatory cytokine production, NET formation and ROS generation [51-53]. On the other hand, ROS were shown to be involved in mTOR pathway regulation and outcome [54]. This ROS interplay with mTOR-centered signaling pathways may promote aging [55, 56]. We can speculate that mTOR pathway is overstimulated in neutrophils in most of elders, in contrast to young people and nonagenarians, resulting in or being accompanied by the increase of intracellular ROS generation in these cells. This overstimulation condition may lead to decreased responsiveness of neutrophils to recurrent activating stimuli because of development of compensatory resistance to the signals that can be a reason for declined ROS response of neutrophils to opsonized zymosan in elder group. We can also suggest that at the stage of signal-resistance the cells use all their reserves to maintain normal cell functioning in the presence of high amount of intracellular ROS. Heat shock proteins executing chaperone functions play in these conditions a role directed to protection of cell signal pathways that can support zymosan-induced ROS response in neutrophils. This may explain the registered in our study positive correlation between the level of zymosan-induced ROS production and all analyzed parameters of intracellular HSP70 in neutrophils of elders (Table).

The absence of the positive HSP70-ROS correlations in neutrophils of nonagenarians may be considered as a factor associated with longer life span of people in this group, along with higher intracellular content of HSP70 associated with more protective ROS response, an important immune function of neutrophils. Conversely, the positive association between intracellular HSP70 and zymosan-induced ROS production could anticipate individuals at a higher risk of mortality prior to their 90th year.

## MATERIALS AND METHODS

### Participants

26 healthy young persons (aged 20-59, 13 men, median 30.5), 24 elders (aged 60-89, 9 men, median 73.5) and 15 nonagenarians (aged 90 and over, 7 men, median 91) registered as patients of the Moscow Clinical Centre of Gerontology were recruited to the study. Inclusion criteria for the participation were the absence of active pathologies (history of acute infection, tumor, apoplexy, myocardial infarction), treatment with corticosteroids or high doses of nonsteroid anti-inflammatory drugs for all subjects and the independent living for elders and nonagenarians. The study has been approved by the local research ethics committee. All participants gave their informed consent prior to the study.

### Neutrophil isolation

Neutrophils were isolated from peripheral blood within 2 h after blood sampling by centrifugation at 500 g for 30 min at RT in density gradient using PolymorphPrep separation medium (Axis-Shield, Sweden). Fractions containing neutrophils were then collected. Cells were washed twice (400 *g*, 15 min) in Dulbecco's phosphate buffer saline (DPBS), resuspended in RPMI-1640 media (Sigma-Aldrich, USA) supplemented with 2 mM *L*-glutamine, 15 mM HEPES and 2% fetal calf serum (HyClone, Thermo Scientific, USA) (referred hereafter to as assay media) at concentration of 2×10^6^ cells/ml and left for 30 min prior to use in assays. Routinely, neutrophil purity was assessed by flow cytometry analysis and was routinely found to be ≥ 95%. Cell viability determined by trypan blue staining was no less than 97%.

### Measurement of spontaneous intracellular ROS production

Spontaneous intracellular ROS generation (ROS_DCF spont_) in neutrophils was determined using 2′-7′-dichlorodihydrofluorescein diacetate (DCFH-DA, Invitrogen, USA) [37]. The probe was added to neutrophils resuspended in assay media (500 μl) at 5 μg/ml final concentration. After incubation for 20 min at 37°C cells were washed twice with DPBS at 4°C. Fluorescence at 530 nm was then measured in neutrophils by flow cytometry on BD FACSCalibur flow cytometer (San Jose, CA) with excitation at 488 nm.

### ROS measurement by luminol-amplified chemiluminometry

ROS production was assessed by luminol-amplified chemiluminometric method [30]. Neutrophils (2×10^5^ cells/sample) were stimulated with zymosan A (Sigma-Aldrich, USA) opsonized with a freshly prepared serum pool of 10 donors at a final concentration of 20 mg/ml to induce ROS production. The reaction was carried out at 37°C in plastic tubes in colorless Hank's Balanced Salt Solution (HBSS) (200 μl) and 1 μM luminol (Serva, Germany) in a volume 400 μl. The level of chemiluminescence in the cell samples was measured by using 3603 chemiluminometer (Dialog Joint Venture, Russia). Numbers of light pulses per minute (cpm) were registered. The kinetics of the level of chemiluminescence was recorded for 30 min. Spontaneous ROS production was measured before zymosan treatment as initial count per minute (cpm) level. For calculation of zymosan-induced ROS production in a sample the maximal cpm level was used. Experiments were performed in duplicate.

### Heat treatment and intracellular HSP70 immunolabeling

Neutrophils in assay media were dispensed into polypropylene tubes (10^6^ cells in 500 μl) and heated in a constant-temperature water bath at 40°C for 40 min or at 43°C for 10 min (heat shock). Some of the samples after heat treatment were then incubated at 37°C for different time intervals. Intracellular levels of HSP70 were then determined by indirect immunofluorescent staining followed by flow cytometry analysis. For intracellular labeling the neutrophils were fixed and permeabilized in DPBS containing 2% paraformaldehyde (Riedel-de Haen, Germany), 0.05% BSA and 0.05% Triton X-100 (Sigma-Aldrich, USA) at 37°C for 15 min. The permeabilized neutrophils were treated in 100 μl volume with primary HSP70-specific monoclonal antibody BRM22 (Sigma-Aldrich, USA) or in HSP70-specific B-hybridoma supernatants in 1:100 dilutions for 30 min at RT and then stained with secondary sheep anti-mouse IgG Fab-fragments conjugated with PE (Sigma-Aldrich, USA) for 30 min at RT. Each stage of labeling was followed by two washes with DPBS containing 0.2% BSA and 0.1% Triton X-100. The cells were finally resuspended in DPBS and analyzed by flow cytometry. HSP70 intracellular levels were determined as means of fluorescence intensity (MFI) corrected for background fluorescence of the negative controls.

### Flow cytometry

Flow cytometry analysis was carried out on a FACSCalibur flow cytometer (BD Biosciences, USA) equipped with 488 and 640 nm lasers and an appropriate set of detectors and filters. Neutrophils were identified and gated using forward and side light scatter. A minimum of 10000 gated events was collected for each sample. Data were analyzed using CellQuest ver. 3.4 (BD Biosciences) and FlowJo version 7.6.5 flow cytometry analysis programs.

### Real-time PCR

Total RNA for each time point was extracted from 6х10^6^neutrophils using TRIzol reagent (MRC, UK). All RNA samples were treated with DNaseI (Thermo Scientific, USA) to remove residual DNA. Total RNA was quantified with NanoVue Plus spectrophotometer at A260/A280 (GE Healthcare Life Science, UK). cDNA synthesis was performed using random hexamer primers with addition of MINT Reverse Transcriptase (Evrogen, Russia) following the manufacturer's instructions. The hexamer primers (12 pmole) were annealed in 10 μl of a mixture containing 2 μg total RNA. The mixture was heated for 2 min at 70°C and then chilled on ice for 10 min. To synthesize cDNA, the reaction mixtures were incubated at 42°C and for 70°C for 120 min and 15 min, respectively. Real-time PCR primers (5′-3′) designed with Primer Blast software (www.ncbi.nlm.nih.gov/tools/primer-blast) and controlled with OligoAnalyzer 3.1 program are listed below (gene: forward primer, reverse primer).

*HSPA1*: AGGTGCAGGTGAGCTACAAG, CTCGGCGATCTCCTTCATC;

*HSPA8*: TGCTGCTCTTGGATGTCACT, AAGGTCTGTGTCTGCTTGGT;

*b-actin*: CACCACACCTTCTACAATGAG, GTCTCAAACATGATCTGGGTC.

The specific fragments were amplified and then checked by 3% agarose gel electrophoresis to detect a single product of the expected size.

### Real-time PCR detection

Each real-time PCR mixture (final volume 25 μl) contained 5 μl of qPCRmix-HS SYBR mixture (Evrogen, Russia), 1 μl of 3 mM forward and reverse primers, 0.5 μl of cDNA template, and 17.5 μl of nuclease-free water. The reactions were carried out using a LightCycler 480 Real-time PCR detection system (Roche Diagnostics, Deutschland GmbH) as follows: pre-incubation at 95°C (5`), and then 40 cycles of 95 °C for 10 s, 60°C for 10 s, and 72 °C for 10 s. At the end of the amplification, a dissociation curve was plotted to confirm the specificity of the product. All real-time experiments were repeated in triplicates. The gene expression ratio was estimated as the difference in quantity from samples that treated under the heating 43°C 10 min versus 40°C 40 min. The results were normalized against house-keeping gene b-actin to correct the sample-to-sample variation.

### Statistical analysis

Statistical analysis was performed using SigmaPlot ver. 11.0 (Systat Software Inc.). For data that followed a normal distribution t-tests were performed to estimate differences between two groups. For non-normally distributed data a Mann-Whitney *U* test was used. Correlation analysis was done using Spearman rank order method. Results were considered statistically significant at p ≤ 0.05.
